# Experience of an anatomic femoral stem in a UK orthopaedic centre beyond 20 years of follow-up

**DOI:** 10.1007/s00590-024-03901-1

**Published:** 2024-04-03

**Authors:** G. Turnbull, C. Blacklock, A. Akhtar, E. Dunstan, J. A. Ballantyne

**Affiliations:** 1 National Treatment Centre Fife Orthopaedics, Victoria Hospital, Hayfield Road, Kirkcaldy, KY2 5AH Scotland, UK; 2 The Royal Infirmary of Edinburgh, 51 Little France Cres, Old Dalkeith Rd, Edinburgh, EH16 4SA Scotland, UK

**Keywords:** Stem, Survivorship, THA, Anatomic, Cement, Periprosthetic fracture

## Abstract

**Introduction:**

Increasing interest in the use of anatomical stems has developed as the prevalence of periprosthetic fractures (PPFs) continues to increase. The primary aim of this study was to determine the long-term survivorship and PPF rate of an anatomical femoral stem in a single UK centre.

**Patients and methods:**

Between 2000 and 2002, 94 consecutive THAs were performed using the 170 mm Lubinus SP II anatomical femoral stem in our institution. Patient demographics, operative details and clinical outcomes were collected prospectively in an arthroplasty database. Patient records and national radiographic archives were reviewed finally at a mean of 21.5 years (SD 0.7) following surgery to identify occurrence of subsequent revision surgery, dislocation or periprosthetic fracture.

**Results:**

Mean patient age at surgery was 65.8 years (SD 12.5, 34–88 years). There were 48 women (51%). Osteoarthritis was the operative indication in 88 patients (94%). Analysis of all-cause THA failure demonstrated a survivorship of 98.5% (95% confidence interval [CI], 98.0–99.3%) at 10 years and 96.7% (94.5–98.9%) at 21 years. The 20-year stem survival for aseptic loosening was 100% with no cases of significant lysis found (lucent line > 2 mm) and no stems required revision. Patient demographics did not appear to influence risk of revision (*p* > 0.05). There were 2 revisions in total (2 for acetabular loosening with original stems retained). There were no PPFs identified at mean 21.5 year follow-up and 5 dislocations (5%).

**Conclusions:**

The Lubinus SP II 170 mm stem demonstrated excellent survivorship and negligible PPF rates over 20 years following primary THA.

## Introduction

Since Sir John Charnley’s pioneering work over 60 years ago, total hip arthroplasty (THA) has become established as amongst the most cost-effective interventions in medicine with excellent long-term outcomes [1–3]. However, as the indications for THA have expanded, the clinical burdens of revision total hip arthroplasty (RTHA) and periprosthetic fractures (PPFs) have also increased [4–6]. Both RTHA and PPF are projected to rise significantly over the coming decades, with high associated healthcare costs, morbidity and mortality for patients [7]. The risk of PPFs is thought to be increased in cemented femoral stems where polished taper slip (PTS) implants are used, despite PTS stems otherwise having consistently excellent clinical results [8, 9]. Interest in the use of anatomical and composite beam (CB) stems has therefore increased, as strategies to prevent PPFs gain increasing importance.

**Fig. 1:**
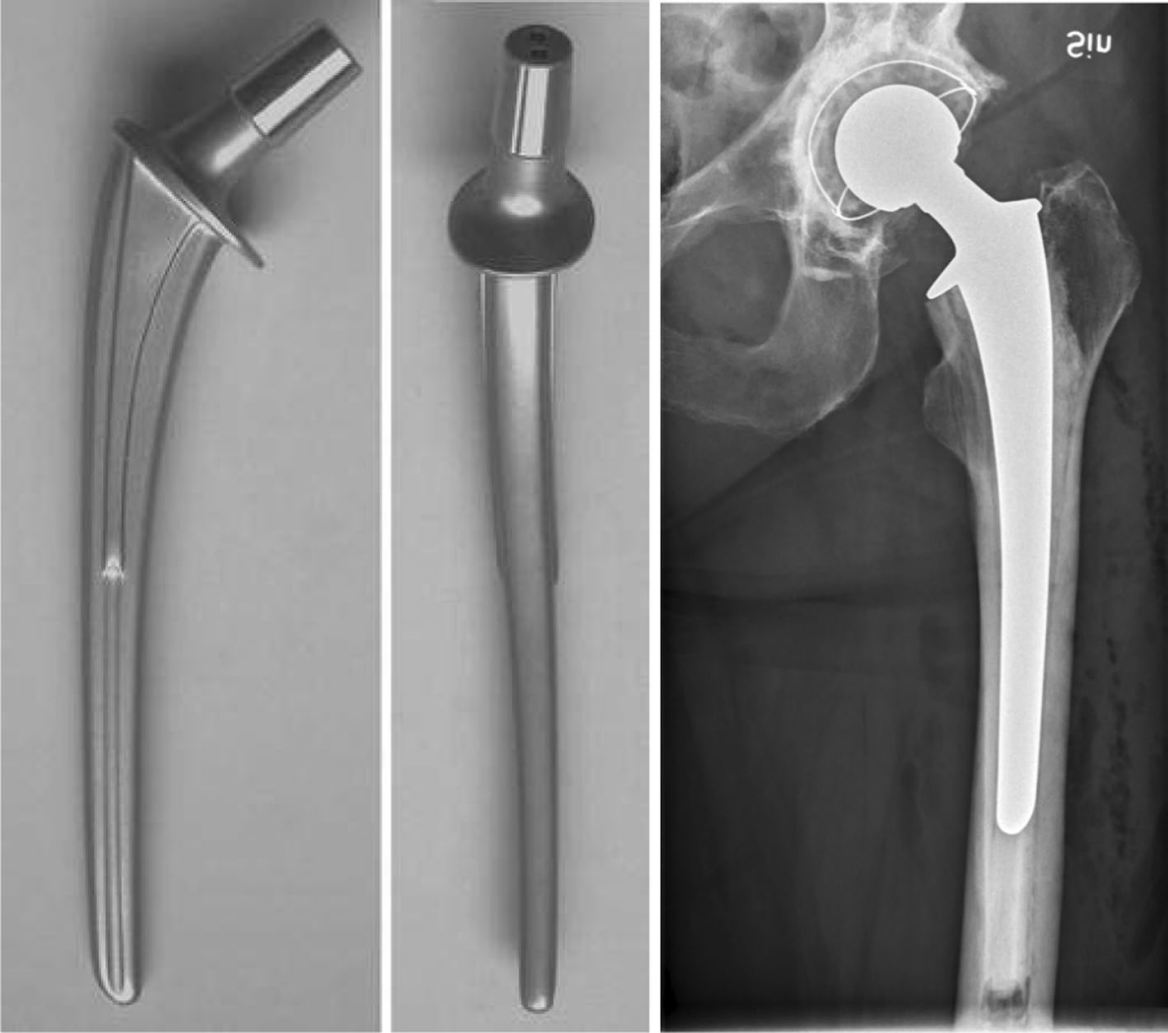
Orthogonal views of the Lubinus SP II stem, demonstrating anatomical geometry in both planes. Example of cemented stem also provided

The Lubinus SP II is an anatomical CB stem (Waldemar Link, Hamburg, Germany) that was first introduced in 1982 with excellent survivorship rates and low periprosthetic fracture rates demonstrated in the Swedish Hip Arthroplasty Register (SHAR) [10]. Composed of cobalt–chromium–molybdenum alloy (Co–Cr–Mo), it has a tapered, anatomically s-shaped stem, with a collar, matte finish and 19° of built-in anteversion of the femoral neck (Fig. 1). The stem is available in 7 sizes (left and right), 3 different lengths (130, 150 and 170 mm) and 3 different caput–column–diaphysis (CCD) angles with corresponding differences in offset (117°, 126° and 135°). The stem collar acts to minimises subsidence [11], while the anatomic shape encourages neutral positioning in the canal, helping provide rotational stability [12]. The anatomic shape also allows for a more even cement mantle thickness to be achieved and lower rates of cement mantle fracture have been found to occur compared to in straight PTS femoral stems [13, 14]. Ultimately, this reduces the risk of cement mantle deficiency developing, which can lead to osteolysis and potential periprosthetic fracture [13, 15–17]. Recently ten times lower rates of Vancouver type B fractures have been observed to occur in the Lubinus SP II as compared to the Exeter stem in the SHAR [18].

Uptake of the stem remains more limited out with Scandinavia where other femoral stems (often with contrasting stem design philosophies but excellent results) have risen to prominence [8, 9, 19]. The primary aim of this study was therefore to determine the long-term survivorship and PFF rate of the Lubinus SP II in a single United Kingdom (UK) centre with two decades experience of implanting it.

## Patients and methods

Between 2000 and 2002, 94 consecutive primary THAs incorporating a 170 mm Lubinus SP II femoral stem were performed within our institution. These patients were identified from a prospectively compiled arthroplasty patient database administered by a dedicated audit nurse. Pre-operative data were collected prospectively including patient demographics, body mass index (BMI), ASA grade (American Society of Anaesthesiologists) and Harris Hip Score (HHS). The HHS is an extended hip function evaluation, which assesses the patient’s perception of pain, function, ability to undertake activities and range of hip motion. The score ranges from 0 to 100, with higher scores indicating increased perceived success and satisfaction [20]. The Scottish Index of Multiple Deprivation was used to assign social deprivation scores to patients based upon postcode. The SIMD ranks geographic areas based upon seven domains: income, employment, education, housing, health, crime and geographical access. Data zones are defined by postcodes and once ranked nationally are divided into population-weighted quintiles with 1 representing the most deprived and 5 the least deprived [21].

Postoperatively, the operating consultant submitted intraoperative data detailing surgical approach, head size and components used. Patients were reviewed 6 weeks postoperatively in an orthopaedic clinic by the operating consultant. They were then followed up at a dedicated orthopaedic audit clinic by two specialist nurses up to 10 years postoperatively and data collected prospectively.

All patient records (including deceased patients) and national radiographic archives were reviewed again finally at a mean of 21.5 years (SD 0.7) following surgery to identify occurrence of mortality, revision surgery, dislocation or periprosthetic fracture at any time following original surgery. Data in presented analyses were derived from patients either up to point of their death or final follow-up.

## Surgical technique

All of the operations were primary, unilateral THAs performed, or supervised, by one of 6 different consultant orthopaedic surgeons with an interest in lower limb arthroplasty. All patients underwent surgery in a lateral decubitus position in a theatre with laminar flow; a modified Hardinge or posterior approach was used according to surgeon preference. One hundred and seventy millimetre Lubinus SP II femoral components were used throughout. Pre-operative templating with calibrated images was performed to aid implant selection, with adjustment made intraoperatively as needed to achieve a balanced hip. After broaching and lavage of the femur, a Hardinge cement restrictor was inserted. A third-generation cementing technique was performed involving pulsatile jet lavage, retrograde cement application and 3-phase pressurization before Lubinus stem insertion. A similar technique was used to implant cemented acetabular components. Cemented Elite Plus components (DePuy Synthes) were used in all acetabula. Twenty-eight millimetre metal heads were used throughout except in 4 patients where 32 mm heads were used. Palacos R & G cement was used for cementation (Heraeus Kulzer GmbH; Heraeus Medical GmbH). All patients had a spinal anaesthetic unless it failed or was contraindicated. Drains were not used. Antibiotic prophylaxis was by a single intravenous dose of 1 g ceftriaxone unless contraindicated, with standardised DVT prophylaxis also given.

## Statistical analysis

This was performed using Statistical Package for the Social Sciences version 28.0 (SPSS Inc., Chicago, Illinois). Univariate analysis was performed using parametric (Student’s *t*-test: paired and unpaired) and non-parametric (Mann–Whitney U test) tests, as appropriate, to assess continuous variables for significant differences between two groups. One-way analysis of variance (ANOVA) was used to compare continuous variables with multiple groups (survivorship in SIMD groups). The Kaplan–Meier method was used to estimate the survival of the prosthesis. A *p* value of < 0.05 was considered significant in all analyses.

## Results

### Demographics

At mean of 21.5 years (SD 0.7) following surgery, 52 patients had died (52 THAs) with 37 patients still alive (42 THAs) at final follow-up. Mean patient age at surgery was 65.8 years (SD 12.5, 34–88 years). There were 48 women (51%). Osteoarthritis was the operative indication in 88 patients (94%), rheumatoid arthritis in 2 patients (2%) and other diagnoses in 4 patients (4%). The mean body mass index (BMI) at surgery was 28.6 kg/m2 (range 18 to 52, SD 5.8).

### Hip scores

Mean HHS was found to significantly improve 1 year following surgery (91 v 45 *P* < 0.001), with maintained improvement seen 10 years following surgery (88 v 45, *P* < 0.001).

### Implant survival

**Fig. 2:**
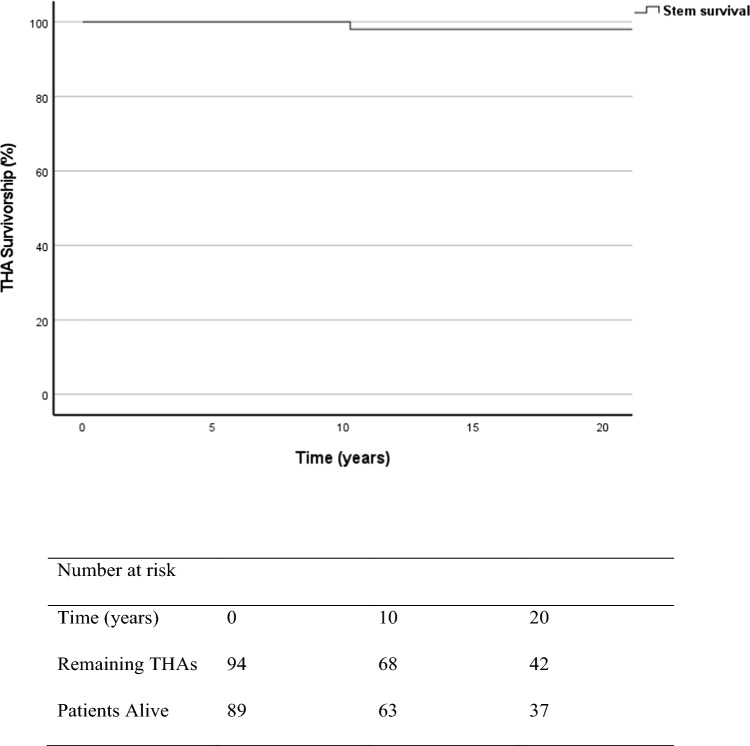
All-cause THA survivorship analysis and stems at risk at selected time points

Analysis of all cause THA failure demonstrated a survivorship of 98.5% (95% confidence interval [CI], 98.0–99.3%) at 10 years and 96.7% (94.5%–98.9%) at 21 years (Fig. 2), with results comparing favourably to many of the most frequently used stems in the UK (Table 1). The 20-year stem survival for aseptic loosening was 100% with no cases of significant lysis found (lucent line > 2 mm) and no stems required revision. There were 2 revisions in total (2 for acetabular loosening with original stems retained). No significant difference was found in implant survivorship comparing patient sex (*P* = 0.2), BMI (*P* = 0.18), SIMD (*P* = 0.34), operative indication (*P* = 0.45) or ASA class (*P* = 0.46).

**Table 1:** Published survivorship and periprosthetic fracture rates of commonly used femoral stems

Femoral stem	Follow-Up	All-cause survivorship (%)	Stem survivorship aseptic Loosening (%)	Stem philosophy	PPF rate (%)
C-Stem [22, 23]	Minimum 25 years	30 at 30 years 95.8 at 10 years	93 to 95%	PTS	1.4 at 15 years [24]
Corail [25]	23 years	82.5	96	Cementless	1.1 at 21 years [25]
Furlong [26]	22.5	91.7	100	Cementless	1.4 at 22.5 years [26]
Lubinus Sp II [18]	18	97 at 15 years	99 at 18 years	CB	0.3% at 15 years [27]
Exeter Universal Stem [28]	22.8	82.9	99 at 22.8 year	PTS	2.3% at 15 years [24, 29]
Exeter V40 [30, 31]	13.5	91.2 – 96.9	99.85 – 100	PTS	1.5% at 10 years[30]
CPT [8]	15	93.4	99 at 10 years	PTS	3.3% at 15 years [24]
Stanmore [32]	22	85	91	CB	0.7% at 22 years [32]

### Periprosthetic fracture, dislocation and complication occurrence

There were no PPFs identified at mean 21.5 year follow-up and 5 dislocations (5%), with the majority happening in the first 3 post-operative months (4/5, 80%) and no patients required revision for recurrent dislocation. Those that dislocated had undergone Modified Hardinge approach in common with nearly all other patients. Mean BMI was significantly higher in those who experienced dislocation (33 SD 5 v 28 SD 5, *P* = 0.04), while patient sex, age, ASA, head size and SIMD had no significant influence on dislocation rate (*p* > 0.05). A small number of patients also developed superficial wound infections and venous thromboembolic events postoperatively (Table 2.).

**Table 2:** Patient experience of complications

Complication	Number	Comments
PE/DVT	3	Three post-op DVT
Infection	3	Three superficial wound infections treated with antibiotics
Dislocations	5	A total of 5 dislocations (1.5%) identified at mean 21.5 year follow-up, with the majority happening in the first 3 post-operative months (4/5, 80%)
Periprosthetic fracture	0	No periprosthetic fractures identified on national records
Revision THR	2	Two for acetabular loosening

## Discussion

The overall survivorship of the Lubinus SP II stem was excellent at a mean of over 21 years follow-up in our centre. Survival to revision for any reason was above 98% at 10 years and 96% at 20 years, with our data representing the longest follow-up data reported for the SP II stem. This appeared to be achieved irrespective of patient demographics, while patient reported hip scores were also excellent at 10-year follow-up. Significantly, no episodes of PPF were found to occur, despite long term follow-up.

Previous European data have demonstrated excellent survivorship rates for the Lubinus SP II [18, 33–36]. When compared to other commonly used femoral stems in the UK (Table 1), it is apparent that excellent long-term implant survivorship in THA can be achieved using a range of differing femoral stems. However, the low PPF rate of the Lubinus SP II is notable with other stems in popular usage having higher published PPF rates (Table 1). While it is a limitation that only 94 of the 170 mm stems are included in our study, a previous report from our centre including 1 000 of the 150 mm SP II stem found a very low PPF rate of 0.3% beyond a mean of 12 years follow-up [27]. Strategies to help minimize PPF risk in patients are of increasing clinical importance; patients suffering PPF display inferior functional outcomes scores, and are at risk of significant morbidity and mortality with a reported 1-year mortality rate approaching 10% [37]. Future projections suggest PPFs are expected to increase by 4.6% every decade over the next 30 years [7]. PPF rates in PTS stems are recognized as being significantly increased compared to in anatomic CB stems, especially in higher risk populations such as the elderly [33]. Within PTS stems, even higher risk of PPF has been noted in the CPT stem. The narrower shoulder radius of the CPT is thought to act like a wedge, helping to split the femur following a fall [38, 39]. Overall revision rates for PPF in PTS stems such as the Exeter have been reported at 2.3% [29] and 1.5% for the updated Exeter V40 stem at 10 years [30]. While the rate of Vancouver type C fractures was found to be similar between the Exeter and Lubinus SP II stem in a recent analysis of over 80 000 patients from the Swedish Hip Arthroplasty Register (SHAR), the rate of Vancouver type B fractures was found to be 10 times higher in the Exeter stem [40]. Exceptionally, low rates of PPF have been demonstrated in other anatomic femoral stems such as the Olympia (Biomet UK Ltd.) in a UK population [41]. In this context, consideration of the use of anatomic femoral stems in at risk patients therefore seems reasonable as part of a strategy to reduce PPF rates.

The 170 mm stem in this study has subsequently been supplemented by 150 mm and 130 mm stems, and indeed 130 mm stems are now the first choice implant in our centre. Excellent survivorship rates have been demonstrated with all three stem lengths, with universally low PPF rates found [27, 35]. Use of the shorter 130 mm stem has several theoretical advantages, including preservation of bone stock, easier removal at time of revision and better proximal filling around the prosthesis. The 170 mm stem potentially offers greater rotational stability, but in practice no significant difference in survivorship has been demonstrated. In terms of revision surgery, cement-in-cement revision is well described for the SP II [18]. However, the anatomical nature and rotational stability of the stem may predispose it to being harder to remove than equivalent straight PTS stems, and based on our experience is significantly aided by use of the correct stem extractor at time of revision.

Of note the smallest available SP II stem (01 size) has been found to be at slight risk of earlier failure [33]. Within our cohort, the stem was only used three times and so no conclusions could be drawn. The increased risk of failure is thought to result from smaller stems having reduced rotational stability and a smaller contact area. This in turn may increase risk of stem debonding from the cement mantle, leading to increased abrasive wear, particle release and osteolysis. It has been suggested that alternative stems are considered in young active patients with narrow canals to decrease risk of revision [18]. Other factors have also been implicated in increasing the hazard ratio for SP II revision at long-term follow-up, including use of extra offset stems, or use of an extra-long head, with stem numbers in our study limiting similar analysis [18].

The dislocation rate found in our study at long-term follow-up is broadly comparable to those reported in the wider literature, with a meta-analysis of over 13 000 primary THAs and minimum 12-month follow-up reporting a dislocation rate of 3.23% for the posterior [29]. However, we previously found the dislocation rate to be lower when reporting outcomes from the 150 mm stem [27]. This may be reflective of our centre having several years’ experience of implanting the SP II stem when data on 150 mm stems were collected, with our data on 170 mm stems derived from our first three-years’ experience of using the SP II, hinting at a potential learning curve. Nevertheless, patients with a higher BMI were at increased risk of dislocation in both 170 mm and 150 mm stems, a finding replicated in previous studies including a range of femoral implants [42–44]. This is a finding that may be counselled to higher BMI (≥ 30 kg/m^2^) patients preoperatively and may also be addressed surgically by consideration of increased implant constraint [45].

There are some limitations to our findings. In terms of the low PPF rate we found, comparison to a matched UK population receiving a PTS stem would allow examination of how the findings of the SHAR (where risk of PPF was significantly increased in PTS stems) are translated into a UK population. Inclusion of detailed patient reported outcome measures at long-term follow-up would have allowed a more detailed view of the success of the SP II beyond metrics such as survivorship. Despite this, hip specific scores up to 10 years post-op were within the range accepted as excellent for the HHS, and comparable to those reported for the Exeter stem [46]. Our study is single centre by nature (in a unit with over 20-years’ experience of using the SP II), potentially limiting external validity of results. PTS femoral stems remain by far the most frequently implanted stems in the UK with excellent results reported [8, 9, 19, 47]. Given the different philosophy of the SP II compared to PTS equivalents, it may be reasonable to expect some degree of learning curve to impact on results if UK uptake of the implant were to increase. However, reassurance can be gained from the excellent survivorship of the SP II demonstrated at national joint registry level [18, 33, 35]. While every effort was made to capture episodes of dislocation, PPF or revision by searching national radiographic archives, there remains the possibility that some episodes were missed in patients relocating. Furthermore, many patients died of unrelated causes by time of final follow-up, potentially leading to underestimation of revision risk. Analysis of a greater number of patients would therefore add weight to our own findings. However, all follow-up data were included in analyses up to point of patient death.

In conclusion, the Lubinus SP II stem was demonstrated to be associated with an excellent survivorship beyond 20 years in our centre, equal to that of the most popular stems in current usage. This survival rate appears to be achieved irrespective of patient demographics. Furthermore, it has a negligible rate of PPF, which should promote continued use of the implant in future as the clinical burden of PPFs continues to increase.
